# Identifying tumour microenvironment-related signature that correlates with prognosis and immunotherapy response in breast cancer

**DOI:** 10.1038/s41597-023-02032-2

**Published:** 2023-03-03

**Authors:** Hongying Zhao, Xiangzhe Yin, Lixia Wang, Kailai Liu, Wangyang Liu, Lin Bo, Li Wang

**Affiliations:** College of Bioinformatics Science and Technology, Harbin Medical University, Harbin, 150081 China

**Keywords:** Breast cancer, Prognostic markers

## Abstract

Tumor microenvironment (TME) plays important roles in prognosis and immune evasion. However, the relationship between TME-related genes and clinical prognosis, immune cell infiltration, and immunotherapy response in breast cancer (BRCA) remains unclear. This study described the TME pattern to construct a TME-related prognosis signature, including risk factors PXDNL, LINC02038 and protective factors SLC27A2, KLRB1, IGHV1-12 and IGKV1OR2-108, as an independent prognostic factor for BRCA. We found that the prognosis signature was negatively correlated with the survival time of BRCA patients, infiltration of immune cells and the expression of immune checkpoints, while positively correlated with tumor mutation burden and adverse treatment effects of immunotherapy. Upregulation of PXDNL and LINC02038 and downregulation of SLC27A2, KLRB1, IGHV1-12 and IGKV1OR2-108 in high-risk score group synergistically contribute to immunosuppressive microenvironment which characterized by immunosuppressive neutrophils, impaired cytotoxic T lymphocytes migration and natural killer cell cytotoxicity. In summary, we identified a TME-related prognostic signature in BRCA, which was connected with immune cell infiltration, immune checkpoints, immunotherapy response and could be developed for immunotherapy targets.

## Introduction

The incidence of breast cancer (BRCA) ranks first among female malignant tumors, and its mortality has been increasing year by year. Breast cancer is the most common cancer among Chinese women, with more than 1.6 million diagnosed and 1.2 million deaths annually^1,2^. BRCA is a heterogeneous disease with multiple biological phenotypes, unique histological features, different clinical manifestations, and different responses to treatment^3^. Over the past few years, we have improved our understanding of the biological functions, molecular and cellular mechanisms, diagnosis and treatment of BRCA^4,5^. Studies have shown that JAK1 plays a role in the prognosis and immune invasion of BRCA by affecting the infiltration levels of dendritic cells, macrophages, CD4+T cells, neutrophils, and CD8+T cells^6^. However, breast cancer treatment remains challenging because treatment options are largely limited to surgery and radiotherapy, and immunotherapy is clinically active in only a minority of breast cancer patients^7,8^. Tumor microenvironment is associated with breast cancer proliferation and immune system suppression as well as clinical treatment^9^.

Tumor microenvironment (TME) refers to the cellular environment in which tumor cells, immune cells, stromal cells and other non-cancerous cells exist^10^. The interaction between malignant and non-malignant cells in TME can influence the development and progression of cancer^11,12^. Tumor-associated immune cells have complex functions in the tumor microenvironment. Anti-tumor immune cells have tumor antagonism, targeting and killing cancer cells in the early stages of tumor development, but cancer cells can suppress tumor antagonism of immune cells through a variety of mechanisms^13^. Tumor-associated stromal cells recruit tumor cells and tumor promoting cells by secreting tumor promoting factors such as IL-6 and IL-8^14^.

Due to the drug resistance and instability of the genome structure of cancer cells, targeting TME offers an opportunity for cancer therapy. Proliferative signaling, cell death, angiogenesis, etc. depend on cancer markers of TME, which have significant advantages in the course of cancer therapy^15^. Tumor-infiltrating lymphocytes (TIL) have been found to significantly affect the 5-year survival rate of non-small cell lung cancer (NSCLC) and have been identified as a prognostic indicator of early-stage NSCLC. This association has led to the use of immune checkpoint inhibitors and improved immunotherapy to treat NSCLC more effectively^16,17^. CXCL5 is an important chemokine in TME, and CXCL5 overexpression is closely related to survival time, recurrence and metastasis of liver cancer patients^18^. Studies have shown that blocking CXCL5/CXCR2 signaling can improve the sensitivity and effectiveness of immunotherapy and slow tumor progression^19^. Therefore, fully understanding the role of TME in the occurrence and development of BRCA and identifying microenvironment-derived biomarkers are of great significance for improving the treatment and prognosis of BRCA patients^20^.

In the present study, we integrated transcriptome information from multiple cohorts of BRCA and systematically analyzed the TME pattern to recognize TME-related genes in BRCA. Then, using univariate Cox regression analysis, multivariate Cox regression analysis and LASSO Cox regression analysis, we established the TME-related prognostic signature in BRCA composed of 6 key TME-related genes and validated them in other datasets. Key TME-related genes synergistically reshape the immune microenvironment of breast cancer and influence the prognosis and immunotherapy effect of BRCA patients. The TME-related prognosis signature is significantly associated with immune cells, clinic-pathological features and somatic mutations of BRCA. As a result, TME-related prognosis signature we identified is a reliable independent prognostic factor and biomarker for BRCA immunotherapy response and prognosis.

## Results

### Identifying tumour microenvironment-related genes in BRCA

The tumor microenvironment (TME) in TCGA-BRCA cohort which was represented by infiltration of immune cells and stromal cells was established using the ESTIMATE algorithm. We found that estimate scores and stromal scores of BRCA samples were distributed at the significantly lower side, compared with those of the normal samples (Wilcoxon rank sum test, P < 0.05), as was immune scores but not significantly (Supplementary Fig. 1a,b). All scores tended to be negatively correlated with tumor grade. Particularly, the stromal scores were greatly related with lymph nodes (Kruskal-Wallis test, P = 0.032), tumor size (P = 0.011), and tumor stage (P = 0.0072). Moreover, with the progression of tumor size, the estimated score decreased significantly (P = 0.044; Supplementary Fig. 2). Although there was no statistically significant difference in the immune score from the aspect of TNM staging system, the immune score showed a decreasing trend with the development of tumor. Furthermore, Kaplan–Meier survival analysis demonstrated that higher immune scores were significantly associated with longer overall survival time (Supplementary Fig. 1c), whereas stromal scores and estimate scores have no significant relevance with patient prognosis. These results implied that the immune cells and stromal cells in TME have a strong evidential clinical relevance in BRCA.

**Fig. 1:**
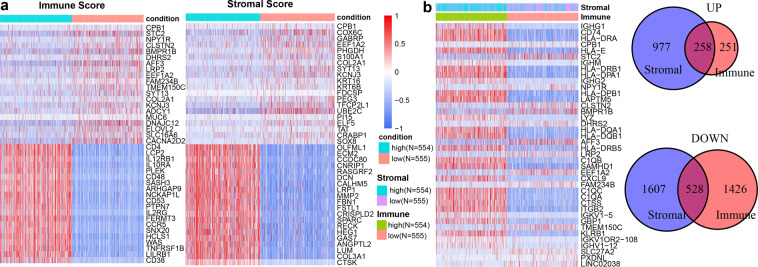
Identifying tumour microenvironment-related genes in BRCA. (**a**) Heatmap of differential expressed genes in the high (N = 554) vs low (N = 555) immune score groups (left-panel) and in the high vs low immune score groups (right-panel). Heatmap of differential genes in the high (N = 554) vs low (N = 555) stromal score groups; (**b**) Heatmap of common differential expressed genes in both stromal and immune groups (left-panel). Venn plots showing common up-regulated and down-regulated DEGs shared by immune score and stromal score (right-panel).

To recognized TME-related genes, the 1109 BRCA samples were classified into high-level groups (N = 554) and low-level groups (N = 555) according to the median immune or stromal score, respectively. We used the DEseq2 package to identify differential expressed genes (DEGs). We identified 509 up-regulated genes and 1954 down-regulated genes based on immune scores (Fig. 1a; Supplementary Fig. 3a). For example, CD38 is up-regulated in samples with high immune scores and is a transmembrane glycoprotein expressed in the immune system and plays an essential role in the treatment of multiple myeloma^21^. CACNA2D2 is up-regulated in low immune samples, and overexpression of CACNA2D2 can promote cell proliferation and angiogenesis, thus promoting tumorigenesis^22^. Similarly, based on stromal scores, 1235 up-regulated genes and 2135 down-regulated genes were obtained (Fig. 1a; Supplementary Fig. 3b). SOX8 is up-regulated in low immune score samples that has a significant effect on cell migration and apoptosis in triple negative breast cancer, and is involved in the maintenance to stem-like capacities in cancer cells^23^. ECM2 is up-regulated in high immune score samples, which could promote the formation of B lymphopoiesis^24^. Next, we have carried out gene ontology (GO) and KEGG enrichment analysis on immune score difference genes and stromal score difference genes through the package “clusterProfiler” and “enrichplot”^25^. Immune score difference gene and stromal score difference genes enrichment presents functional consistency. The results showed that enriched GO of differential genes mainly existed in complement humoral phagocytosis, adaptive immunity based built, antigen response-activating receptor-mediated surface, leukocyte cell-cell proliferation adhesion, and immunoglobulin production molecular mediator. The results showed that KEGG enrichment analysis of differential genes were more concerned with Antigen adhesion arthritis infection, Inflammatory Th1 bowel differentiation, Allograft Autoimmune Graft-versus-host diabetes, Malaria immunodeficiency killer cytotoxicity, and Chemokine Cytokine-cytokine interaction cytokine (Supplementary Fig. 4a–d). A total of 786 common DEGs in both stromal and immune groups, consisting of 258 upregulated genes and 528 downregulated genes were regarded as tumor microenvironment-related genes (TME-related genes; Fig. 1b). Among them, 35 (4.4%) genes overlapped with cancer associate genes (P = 0.0008; hypergeometric test; Supplementary Fig. 4e), which collected from four public databases including the Online Mendelian Inheritance in Man (OMIM) database, HuGE Navigator, PharmGKB, and Comparative Toxicogenomics Database (CTD). And 17 (2.1%) TME-related genes overlapped with BRCA associate genes (P = 0.015; hypergeometric test; Supplementary Fig. 4f). For example, FGF4 is upregulated in most samples with low immune score and low stromal score, and FGF4 gene deregulation affects the overall survival rate of patients with bladder cancer^26^. AGTR1 is upregulated in most of the samples with high immune and stromal scores and promotes lymph node metastasis in BRCA^27^.

### Identifying key TME-related genes as a prognostic signature in BRCA

**Fig. 2:**
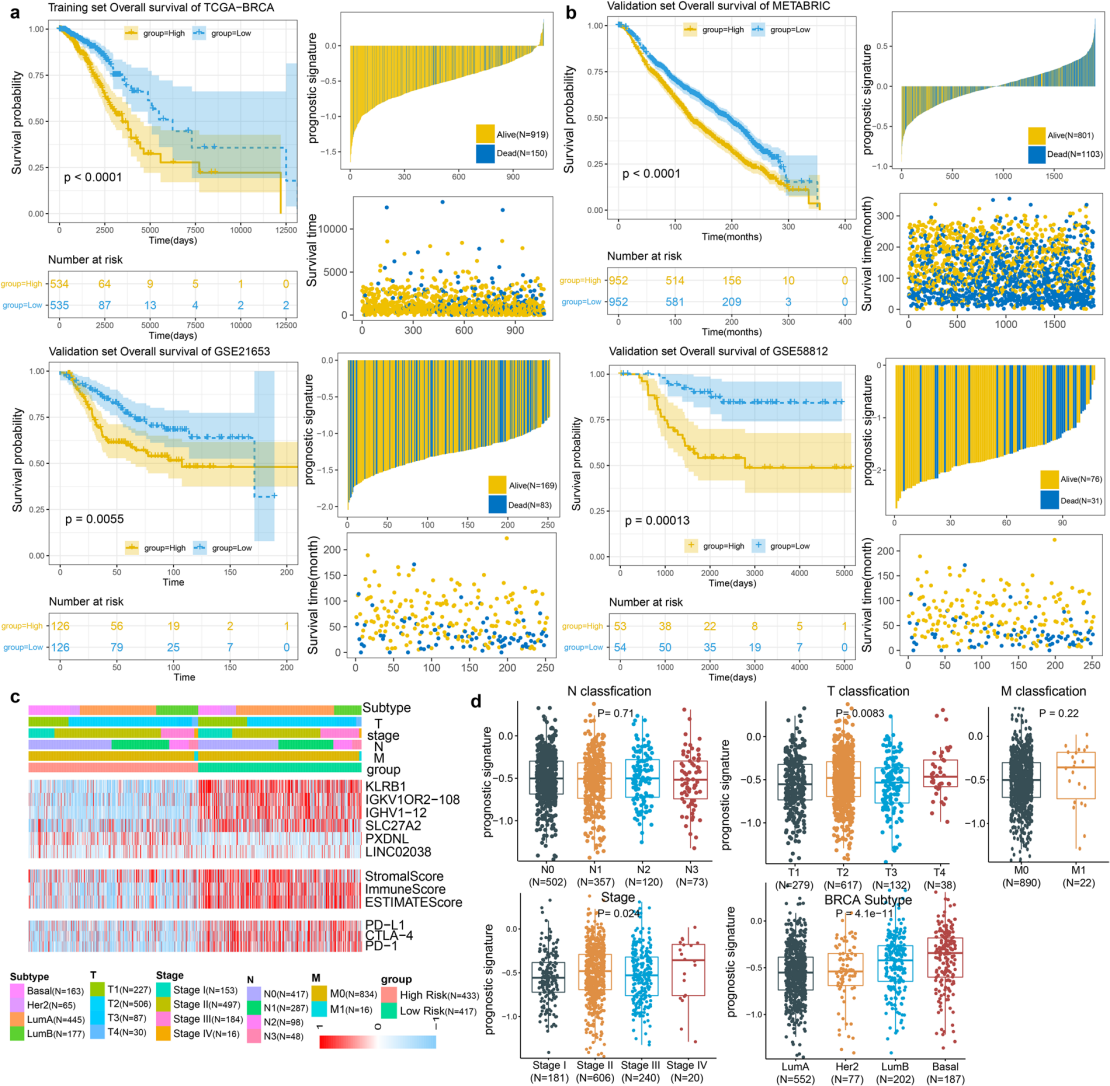
key TME-related genes as a prognostic signature in BRCA. KM survival curves and risk score of OS based on risk scores of the prognostic signature in (**a**) TCGA-BRCA (High = 534, Low = 535) training cohort and in validation cohorts (**b**) including METABRIC (High = 952, Low = 952), GSE21635 (High = 126, Low = 126) and GSE58812 (High = 53, Low = 54). (**c**) Cluster heat map of TME-related genes, immune scores/stromal scores/ESTIMATE scores and immune checkpoints PD-L1, PD1 and CTLA4 stratified by the prognostic signature in TCGA-BRCA cohort. (**d**) Differences in risk scores of the prognostic signature between clinic-pathological feature and BRCA subtypes. Significance P values were calculated using Kruskal-Wallis test.

In order to identify the potential prognostic marker genes associated with the progression of breast cancer. Through univariate Cox regression analysis, 168 TME-related genes were identified as important factors affecting the survival of BRCA patients. After multivariate Cox regression analyses with clinic-pathological characteristics (age, sex, pathological T stage, pathological N stage, pathological M stage and pathological tumor stage), 33 of 168 TME-related genes with FDR < 0.05 were identified as candidate genes associated with prognosis in BRCA. Subsequently, to construct the prognostic feature based on candidate genes, LASSO Cox regression analysis was implemented to determine 6 key TME-related genes, including PXDNL, LINC02038, SLC27A2, KLRB1, IGHV1-12 and IGKV1OR2-108. We then established the comprehensive risk score consisting of 6 key TME-related genes as the TME-related prognostic signature in BRCA. The risk score was calculated as follows: expression of PXDNL * 0.1138 + expression of LINC02038 * 0.0782 + expression of SLC27A2 * (−0.1093) + expression of KLRB1 * (−0.2147) + expression of IGHV1-12 * (−0.1314) + expression of IGKV1OR2-108 * (−0.0029). Since the level of immune infiltration and cellular composition are closely related to tumor progression and patient prognosis, we used the median risk score to classify BRCA samples into high-risk and low-risk groups. The Kaplan-Meier survival curves indicated that BRCA patients in high-risk score group was significantly associated with poor prognosis (Log-rank test, P < 1.0e-4; Fig. 2a). To verify the predictive capacity of the prognostic markers, we evaluated its performance in three independent validation cohorts, including METABRIC (1904 BRCA samples), GSE58812 (107 BRCA samples) and GSE21653 (252 BRCA samples) cohorts. Consistent with the finding in the TCGA-BRCA cohort, we found that the prognostic signature worked well and high-risk score groups were associated with a poorer prognosis in all independent data sets (METABRIC, Log-rank test, P < 1.0e-4; GSE58812, Log-rank test, P = 1.3e-4; GSE21653, Log-rank test, P = 5.5e-3; Fig. 2b). These findings indicate that the prognostic signature has robust predictive potential for BRCA in the training and validation cohorts. Next, we found that the downregulation of KLRB1, IGHV1-12, IGKV1OR2-108 and SLC27A2 and upregulation of PXDNL and LINC02038 were shown in high-risk score group. We also obtained two additional independent validation cohorts from the ICGC database (99 BRCA samples) and Krug, Karsten *et al*. (122 BRCA samples), in which three genes (SLC27A2, PXDNL and KLRB1) were detected at mRNA expression levels. The expressions of SLC27A2 (P = 3e-6; P = 1.2e-3) and KLRB1 (P = 0.046; P = 9.7e-10) were significantly increased in the low-risk group, and PXDNL was significantly increased in the high-risk group (P = 2.9e-12; P = 0.017; Supplementary Fig. 5a,b). Moreover, the immune scores, stromal scores and estimate scores of the high-risk score group were lower than those of the low-risk score group, indicating high infiltration of immune cells and stromal cells in low-risk score group (Fig. 2c). Specifically, immune checkpoint markers, including programmed death 1 (PD-1), programmed death ligand 1 (PD-L1), and cytotoxic T lymphocyte-associated antigen 4 (CTLA-4) were expressed at higher levels in the low-risk score group, indicating that tumor samples with the low-risk score may tend to have favorable responses to anticancer immunotherapies. To further explore how key TME-related genes change at the protein level, we obtained proteomic data PDC000173 from 105 BRCA samples, in which 3 of 5 coding genes (including SLC27A2, PXDNL and IGKV1OR2-108) were captured at the protein expression level^28^. SLC27A2 showed significantly higher protein expression levels in the low-risk group (P = 1.5e-11). And PXDNL displayed higher protein expression levels in the high-risk group (P = 4.4e-8). We found that the differences of key TME-related genes at the protein level and mRNA level were consistent (Supplementary Fig. 5c). In order to characterize whether these genes are regulated by methylation levels, we downloaded illumina methylation 450 K beadChip data of 890 breast cancer samples from TCGA project, and 4 genes (PXDNL, SLC27A2, KLRB1 and IGHV1-12) were detected. We found the methylation levels of 3 genes (PXDNL, SLC27A2, and IGHV1-12) were significantly negatively correlated with mRNA expression levels (Pearson correlation test; P < 0.05, Supplementary Fig. 5d), which suggested these genes are regulated by methylation.

We discovered that risk score significantly increased in the subtypes of basal-like and luminal B which was distinguished by high malignancy and worse prognosis (Kruskal-Wallis test, P < 0.001) and decreased in the low malignancy subtype of luminal A^29^. The risk score was observed to be significantly increased in the breast cancer patients who present with stage II and IV (Kruskal-Wallis test, P = 0.024), and with large tumor size (T4; Kruskal-Wallis test, P = 8.3e-3; Fig. 2d). Moreover, we also found that high-risk group was concentrated on the high malignancy subtypes and high tumor stages, which was consistence with above findings. And high-risk score groups were significantly associated with a poorer prognosis for BRCA subtypes (Supplementary Fig. 6a–d), particularly in LumA (P = 1.2e-4) and LumB (P < 0.0001). It suggests the risk scores of the prognostic signature increased with the development of the malignant phenotype of the tumor and have potential clinical applicability in BRCA.

In addition, we explored whether these six genes are also prognostic markers in other cancer types. We obtained expression and clinical data from the TCGA repository for 32 different cancer types, including 8980 patients. Univariate Cox regression analysis was performed to evaluate the association between survival and expression levels of each of the six genes. A risk score formula was developed to evaluate the association between survival and expression in a certain cancer. Then the median risk score was used as cut-off to classify patients into high and low-risk groups. Moreover, we conducted Kaplan-Meier curves to evaluate the impact of risk score on patients’ overall survival time. The results showed that these 6 key TME-related genes could be used as prognostic signature in 13 cancers (Supplementary Fig. 7), including adrenocortical carcinoma (ACC, P = 1e-04), cervical squamous cell carcinoma and endocervical adenocarcinoma (CESC, P = 8.5e-03), esophageal carcinoma (ESCA, P = 0.042), head and neck squamous cell carcinoma (HNSC, P = 0.037; Supplementary Fig. 7a), kidney renal clear cell carcinoma (KIRC, P < 1e-04), brain lower grade glioma (LGG, P < 1e-04), Mesothelioma (MESO, P = 0.021), pheochromocytoma and paraganglioma (PCPG, P = 4.8e-03; Supplementary Fig. 7b), rectum adenocarcinoma (READ, P = 0.033), sarcoma (SARC, P = 8.6e-04), skin cutaneous melanoma (SKCM, P = 1e-03), thyroid carcinoma (THCA, P = 0.013; Supplementary Fig. 7c), and uterine corpus endometrial carcinoma (UCEC, P = 2.7e-03; Supplementary Fig. 7d). We found that the prognostic signature worked well and high-risk score groups were associated with a poorer prognosis in 13 TCGA cancer types. These results demonstrate the prognostic significance of these six genes, including PXDNL, LINC02038, SLC27A2, KLRB1, IGHV1-12 and IGKV1OR2-108, in several human cancers.

### The prognostic signature acting as an independent prognostic factor in BRCA

To confirm whether the prognostic signature are independent prognostic factors for BRCA, univariate and multivariate Cox regression analyses were performed on BRCA patients. The risk score of the prognostic signature and other clinic-pathological factors, including gender, age, pathological T stage, pathological N stage, pathological M stage and pathological tumor stage, were used as covariates. The results revealed that the prognostic signature, age and pathological tumor stage were independent risk factors to predict the prognosis of BRCA patients, and indicated that the prognostic signature could serve as an independent prognostic factor for BRCA (Supplementary Fig. 8a). Thus, combined with prognostic characteristics, age and tumor pathological stage, a comprehensive Nomogram information map for clinicians to predict the mortality of BRCA patients was further constructed (Supplementary Fig. 8b). Each patient is given a point for each prognostic factor, and the greater the sum of points, the higher the mortality rate. Furthermore, the calibration curves demonstrated the prediction of BRCA mortality is close to the real probability (Supplementary Fig. 8c). Decision curve analyses (DCA) curves also indicated the nomogram had high clinical predictive potential (Supplementary Fig. 8d). Therefore, the combination of the prognostic signature, age and pathological tumor stage could improve the prognosis evaluation for BRCA.

### The prognostic signature correlated with immune cell infiltration in BRCA

**Fig. 3:**
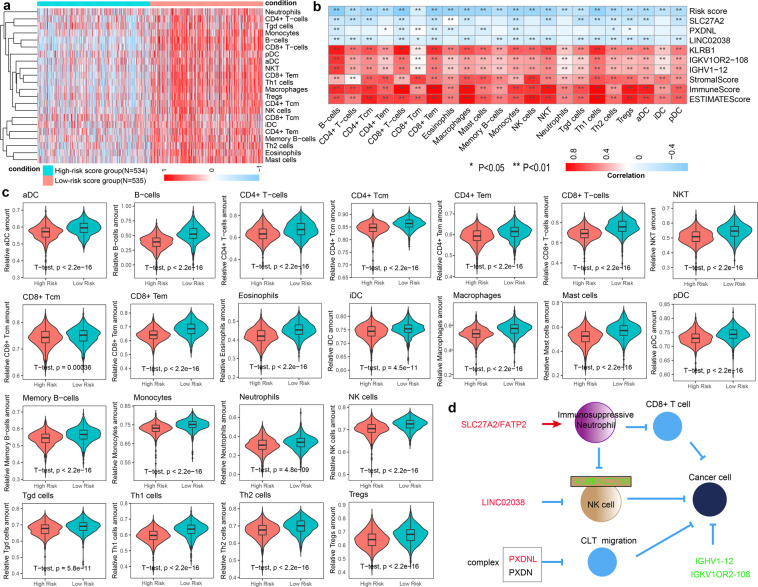
The prognostic signature correlated with immune cell infiltration in BRCA. (**a**) Cluster heat map of abundance of 22 types of immune cells in the high- (N  =  534) and low-risk (N  =  535) score groups in TCGA-BRCA cohort. (**b**) Correlation matrix of risk scores, genes in the prognostic signature, immune score, stromal score, estimate score and the abundance of 22 types of immune infiltration cell. The red indicated positive correlation, while blue indicated negative correlation. (**c**) Violin plots showing the correlation between the prognostic signature and 24 types of immune infiltration cells Significance P values were calculated using Student’s t test. (**d**) Diagram of the prognostic signature and immune system. Red indicates upregulation and blue indicates downregulation of genes in high-risk score group.

To systematically evaluate the differences of the tumor immune microenvironment, xCell was used to quantify the immune infiltration within the tumor based on marker gene sets of 22 distinct leukocyte subsets. We found that patients in high-risk score group had low levels of immune infiltration (Fig. 3a). We also found low levels of immune infiltration in the high-risk score groups of the four breast cancer subtypes, which was consistence with above findings (Supplementary Fig. 6a–d). The prognostic signature showed a significant negative correlation with immune-infiltrating cells, such as natural killer cells (NK cells), the natural killer T (NKT) cells and Neutrophil cells, while immune score, stromal score and ESTIMATE score were related to high infiltration of immune-infiltrating cells (Fig. 3b). We found a significant increase in the proportion of lymphocytes and myeloid cells in the low-risk score group compared with the high-risk score group, such as CD8+ T cells, CD4+ central memory T cells (Tcm), plasmacytoid dendritic cells (pDC), CD8+ Tcm cells, CD8+ effector memory T cells (Tem), NK cells, NKT cells, B cells, Tregs and Neutrophil (Fig. 3c). Moreover, CIBERSORT and TIMER were used to confirm the infiltration of immune cells in tumor samples. We found that immune cells showed more infiltration degree in the low-risk score group, such as CD8+ T cells, B cells, CD4+ T cells and Neutrophil, which was consistent with the above results (Supplementary Fig. 9). The key TME-related genes KLRB1, IGHV1-12 and IGKV1OR2-108 were positively relevant to infiltration of immune cells and LINC02038, SLC27A2 and PXDNL were negatively relevant to infiltration of immune cells (Fig. 3d). The killer cell lectin-like receptor B1 (KLRB1) gene encodes for CD161, a membrane protein of NK cells, showed the downregulated expression in the patients in high-risk score group. Recent studies have shown that CD161 downregulation causes to immune escape in oropharyngeal cancer and is connected with damaged NK cell cytotoxicity in patients with metastatic melanoma^30,31^. IGHV1-12 and IGKV1OR2-108 were downregulated in high-risk score group and both of which are associated with immunoglobulin production by differentiated B lymphocytes^32^. The downregulation of SLC27A2, a fatty acid transporter protein 2 (FATP2) encoding gene, in high-risk score group could mediate suppression of CD8+ T cells by polymorphonuclear myeloid derived suppressor cells or immunosuppressive neutrophils^33–35^. In line with our results, recent studies have shown that SLC27A2-specific inhibitor could substantially delay tumor growth and increase responses to immune checkpoint inhibitors in tumor model mice. Combined with checkpoint inhibitors, SLC27A2 inhibition blocked tumor progression in mice^36^. PXDNL was overexpressed in the BRCA patients in high-risk score group. PXDNL is a novel homolog of PXDN, and PXDNL can form a complex with PXDN, which can impair the cytotoxic T lymphocytes (CTLs) migration and local immunosurveillance^37^. High PXDNL expression is reported to have decreased overall survival or relapse-free survival in breast cancer patients^38^. LINC02038 is often overexpressed in high-risk score group patients and the expression of LINC02038 is negatively correlated with NK cell infiltrates in BRCA. LINC02038 was reported to inhibit the killing effect of NK cells by upregulating expression of TM4SF1^39^.

Then, we performed gene set variation analysis (GSVA) to explore the biological process of genes in the prognostic markers. We found patients in high-risk score group was significantly enriched in E2F targets, MYC targets V1 and MYC targets V2, which are relevant to tumor cell differentiation, proliferation, and metabolism. Similarly, patients in low-risk score group displayed enrichment to IL-6/JAK/STATs pathway, KRAS signaling up, IFN-γ response and IFN-α response, which are associated with the immune activation. Therefore, up-regulated SLC27A2, KLRB1, IGKV1OR2-108 and IGHV1-12 in the low-risk score group synergistically inhibit tumor progression and activating immune response by regulating CD8+ T cells, NK cells and immunoglobulin. Up-regulated PXDNL and LINC02038 in the high-risk score group synergistically construct immunosuppressive microenvironment and promote tumor progression by impairing the cytotoxic T lymphocytes (CTLs) and inhibiting NK cell function. These results suggested that the prognostic signature consisting of 6 key TME-related genes can characterize the changes in the tumor immune microenvironment in the patients with BRCA, and thus influence patient prognosis and immunotherapy.

### The prognostic signature positively correlated with tumor mutation burden

**Fig. 4:**
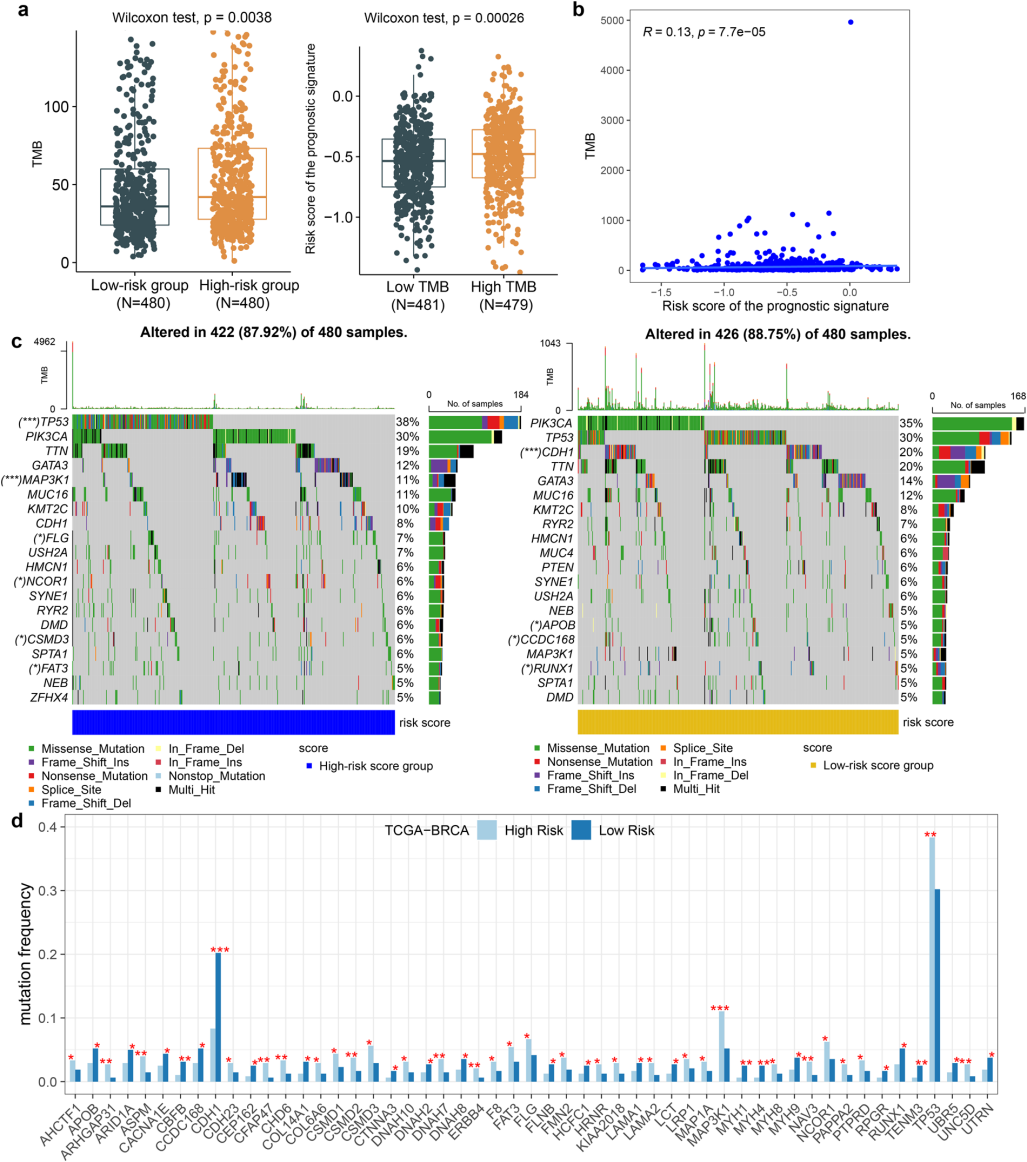
The prognostic signature positively correlated with tumor mutation burden. (**a**) Box plot showing the correlation of TMB with risk score of the prognostic signature. Significance P values were calculated using Wilcoxon rank sum test. (**b**) Scatter plots depicting the spearman correlation between risk score of the prognostic signature and TMB. (**c**) The waterfall plot of tumor somatic mutation displayed distribution of top 20 highly mutated genes in the high-risk (left-panel) and low-risk (right-panel) score groups. (**d**) Significant enrichment of mutant genes in the high and low risk score groups. ***P < 0.001; **P < 0.01; *P < 0.05.

Since somatic mutations are extensively characteristic in BRCA, we describe somatic mutations in breast cancer and explore the relationship between the prognostic signature and tumor mutation burden (TMB). By analyzing the somatic mutations file of TCGA BRCA cohort, we obtained the mutation load, mutation type and mutation distribution of patients. Next, we found that patients in high-risk score group showed higher TMB level (Wilcoxon rank sum test, P = 2.8e-4; Fig. 4a) and higher TMB group showed a higher risk of the prognostic signature (Wilcoxon rank sum test, P = 3.6e-3, Fig. 4a). In addition, there is a significant positive correlation between the prognostic signature and the TMB (spearman correlation analysis, P < 0.001, Fig. 4b). In order to explore the important role of mutated genes between high and low risk groups, we used the permutation test to assess whether mutated genes were enriched in the high- and low-risk groups. We provided the landscape of 53 significantly mutated genes with potential roles in the TME which mutated in at least 1% of the TCGA BRCA samples. Among them, 7.6% mutated genes presented high frequency mutations in BRCA samples (mutation rate>5%; mean mutation rate =  15.5%; Fig. 4c). For example, mutation frequency of TP53 (P = 0.001) and MAP3K1 (P < 0.001) are significantly upregulated in the high-risk score group than in low-risk group. Consistent with our results, oncogene TP53 mutation is a known detrimental prognostic factor in breast cancer patients and MAP3K1 mutations are relevant to shorter survival in metastatic breast cancer^40,41^. Our findings are consistent with previous studies showing that TP53 and MAP3K1 mutations revealed a highly increased risk of breast cancer^42^. In the low-risk score group, the mutation frequency of CDH1 (P < 0.001) is significantly upregulated when comparing with the high-risk group. CDH1 mutated patients exhibited higher immune scores than wild-type patients in breast cancer, which supports our results that the TME of breast cancers correlates with CDH1 mutations^42^. Importantly, the mutation frequencies of 92.4% mutated genes were not high in the BRCA samples (mutation rate < 5%; mean mutation rate = 2.5%), but they showed significantly different mutation frequencies between the high and low risk score groups. For example, the mutation frequencies of ASPM (P = 0.002) and UNC5D (P = 0.006) are significantly upregulated in the high-risk group than in the low-risk group (Fig. 4d). Studies have shown that the protein product of the mutated gene ASPM may have tumor-destroying effects and be a potential therapeutic target for brain tumors^43^. Mutations in UNC5D are involved in the pathogenesis of non-small cell lung cancer by eliminating tumor suppressor functions encoded in proteins^44^. In the low-risk group, the mutation frequency of TENM3 (P = 0.003) and COL14A1 (P = 0.015) are significantly upregulated when comparing with the high-risk group. Studies have shown that mutations in TENM3 are independent predictors of poor survival in esophageal squamous cell carcinoma^45^. COL14A1 is a significant mutant gene associated with the prognosis of gastric cancer, and can predict the survival of patients with newly classified subtypes of gastric cancer^46^. Mutations in these genes may play a potential role in the different TME of breast cancer.

In order to explore the consistency of mutation enrichment of 53 significantly mutated genes in independent validation dataset, METABRIC mutation data were obtained, including 2355 BRCA samples and 173 mutated genes. Among the 53 significantly mutated genes, 16 gene mutations were detected, in which 15 genes showed significant differences in mutation frequency in the high- and low-risk groups. We achieved 93.75% consistency with the independent validation dataset. These results showed that higher risk score of the prognostic signature positively correlated with higher level of TMB in breast cancers, which is a predictive marker for immunotherapy in breast cancer^47^. It suggested that the prognostic signature may also be conducive to predict response to immunotherapy in breast cancers.

### The prognostic signature as a predictive biomarker for immunotherapy in BRCA

**Fig. 5:**
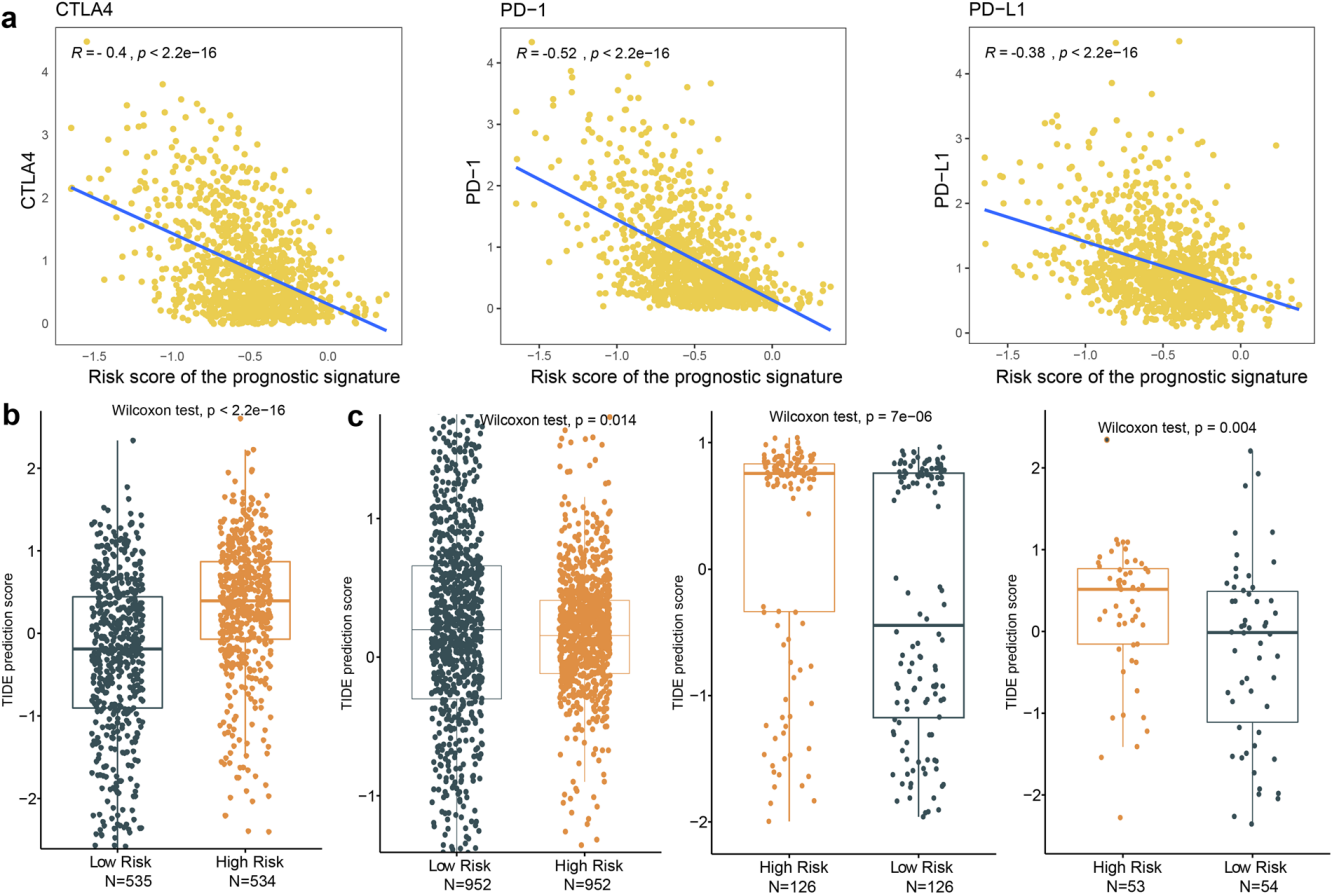
The prognostic signature as a predictive biomarker for immunotherapy in BRCA. (**a**) Scatter plots depicting the negative correlation between risk score of the prognostic signature and the expression of PD-L1, PD-1 and CTLA-4. The spearman correlation is used in calculations. TIDE scores in high-risk and low-risk score groups in (**b**) TCGA-BRCA cohort and (**c**) validation cohorts including METABRIC, GSE21653 and GSE58812. Significance P values were calculated using Wilcoxon rank sum test.

The immune cells infiltrates is connected with clinical outcomes in breast cancers^7^. The expression of immune checkpoints such as PD-1 is used to predict the benefit of immunotherapy in a variety of malignant tumors. PD-1, PD-L1 and CTLA-4 were used as immune checkpoint markers. We constructed the risk score for 6 key TME-related genes as a TME-related prognostic signature to assess the association with immune checkpoints. We discovered that the risk score of the prognostic signature was negatively correlated with the expression of PD-L1, CTLA-4 and PD-1 (P < 2.2e-16; Fig. 5a). PD-L1, PD-1 and CTLA-4 are expressed at higher levels in the low-risk score group compared with the high-risk score group, suggesting that tumor samples with the low-risk score may tend to have favorable responses to anticancer immunotherapies. Furthermore, we sought to investigate whether prognostic characteristics could predict response to immunotherapy as well as prognosis in patients with BRCA. We used tumor immune dysfunction and exclusion (TIDE) algorithm to predict the potential immune response in TCGA-BRCA cohorts^48^. Our results revealed higher TIDE scores in the high-risk score group in BRCA (Fig. 5b), which suggests that immune checkpoint blockade is less effective in patients with high-risk scores that have prognostic signature. TIDE scores were also found to be higher in high-risk score groups of breast cancer Basal, Her2, LumA and LumB subtypes (Supplementary Fig. 6a–d). In addition, we also obtained additional validation sets including METABRIC, GSE58812 and GSE21653 cohorts to investigate predictive ability for immunotherapy using the prognostic signature. The results of the validation cohort showed significant differences in TIDE scores between high-risk and low-risk scores for prognostic markers (Wilcoxon test; Fig. 5c). Our results suggest that the prognostic signature consisting of 6 key TME-related genes could act as a predictive biomarker for immunotherapy in BRCA. Finally, we constructed an online data portal that provides the expression and prognosis of TME-related genes and the relationship between TME-related prognostic signature, TIDE scores, TME, and clinical features (http://tmerpsmap.bio-database.com/).

## Discussion

At present, advances in RNA sequencing technology and the invention of numerous analytical methods have advanced the understanding of tumor pathogenesis. Transcriptome profiling patterns reveal cellular functional states and cellular behavior in relation to genomic and environmental changes^49–51^. Since TME is a cellular environment that includes a variety of cells such as tumor cells, more and more research has turned to TME^52,53^. Therefore, more and more evidences suggest that TME has a critical role in the pathogenesis of BRCA. TME not only interacts with tumor cells to promote their proliferation, but also affects the treatment process^54^. Considerable effort has been invested in exploring the complex mechanisms of BRCA, but the current understanding of TME, therapeutic targets, and prognostic factors remains unsatisfactory^55–58^.

In this study, we first constructed the TME of BRCA and applied LASSO regression analysis to establish TME-related prognostic signature that best represented the characteristics of TME. To identify the robustness of the 6-gene-combination, we compared our method with other six machine learning algorithms, including random survival forest (RSF), Ridge, elastic network (Enet), stepwise Cox, partial least squares regression for Cox (plsRcox), supervised principal components (SuperPC), and the average consistency rate is 75.00% (Supplementary Table 2, 3). We established risk score for each sample and validated our risk score in other data sets and found that patients in the high-risk group had a worse prognosis. Then, to benefit clinical applications, we constructed the nomogram and verified the prediction potential of this model. We also carried out a further study on immune-infiltrating landscapes and immunotherapy response on account of TME-related prognostic signature. In addition, we also studied the effect of somatic mutations on TME. Indeed, our study proclaimed that the TME-related prognostic signature we identified has good prognostic potential.

Among TME-related prognostic signature, KLRB1, SCL27A2, PXDNL, LINC02038, IGHV1-12, IGKV1OR2-108 were directly related to tumor immunity to a certain extent, reflecting the characteristics of natural killer cells, natural killer T cells and other immune cells. For example, the expression of KLRB1 inhibits the cytotoxicity of natural killer cells (NK) and is associated with superior outcome, largely reflect tumor-associated leukocytes. KLRB1, as a membrane protein, can promote IFNγ secretion by NK cells and NKT^59–61^. Recently, a study revealed that the growth of ESCC cells with KLRB1 knockdown was inhibited. In our study, the expression of KLRB1 was positively relevant to the abundance of immune cells, and was highly expressed in the low-risk group, which was a beneficial survival predictor of BRCA. Peroxidasin-like (PXDNL) is mainly expressed in the immunoglobulin I-set domain of major histocompatibility complex (MHC) class I and II and programmed cell death protein 1 (PD1)^62,63^. Some studies have found that PXDNL was highly expressed in breast cancer and affects the survival of patients. We found that PXDNL was highly expressed in the high-risk group and was negatively correlated with many immune cells. SLC27A2 is at work in neutrophil degranulation and mediates neutrophils secretion of cytokines and other inflammatory mediators. SLC27A2 has been found to be under-expressed in many tumors, such as ovarian and lung cancers, and is associated with low survival and chemotherapy resistance^64–67^. We found that SLC27A2 was also up-expressed in the low-risk group and inhibited the immune effect of neutrophils. LINC02308 was significantly overexpressed in glioma and acts as a sponge to express miR-30e-3p to up-regulate TM4SF1 and promote glioma occurrence^68^. In our study, the expression of TM4SF1 was low in the low-risk group and negatively relevant to NK cell. IGHV1-12 and IGKV1OR2-108 are both immunoglobulin-related genes. Immunoglobulin is an immunoactive factor that is crucial in the immune system^69,70^. In our study, IGHV1-12 and IGKV1OR2-108 were positively connected with abundance of immune cells and overexpressed in the low-risk group. Therefore, the up-regulated key TME-related genes SLC27A2, KLRB1, IGKV1OR2-108 and IGHV1-12 in the low-risk score group inhibit tumor progression by affecting the secretion of neutrophils, NK cells and immunoglobulin. In the high-risk score group, up-regulated PXDNL and LINC02038 promote the formation of immunosuppressive microenvironment and promote tumor progression by affecting NK cells and CTL. These results demonstrate that key TME-related genes we identified are synergistically participate in the remodeling of the immune microenvironment and influence patient prognosis, which may be potential therapeutic targets for BRCA. Although immune checkpoint blockade (ICB) therapy has shown remarkable success in treating patients with BRCA and many other types of cancer, only a subset of patients experience long-term benefits and achieve durable clinical responses^71,72^. The lack of effective clinical tools to assist ICB therapy not only results in an inability to classify patients, but its overuse may have substantial side effects and costs^73^. Therefore, it is of great interest to identify biomarkers that predict response to ICB therapy to optimize treatment decisions. In this work, we analyzed the immune infiltration landscape of BRCA on the strength of TME-related genes, and we discovered that the low-risk group had a higher level of immune cells. For the GSVA based on the TME-related prognostic signature, we observed that targets with immunosuppressive and harmful effects on patients, such as E2F target, MYC target V1, and MYC target V2, were enriched in the high-risk population^74^. Moreover, the interferon gamma response and interferon alpha response was enriched in the low-risk group, indicating beneficial survival and immunotherapy responses^75^. We used TIDE algorithm to predict patient response to cancer immunotherapy and found that patients in the low-risk group had a higher proportion of immune responses than those in the high-risk group. Moreover, ICB therapy involves targets including PD-L1, PD-1 and CTLA4^76^. We detected that PD-L1, PD1 and CTLA4 were highly expressed in low-risk group, and were significantly negatively relevant to TME-related genes. These results may account for the better prognosis of patients in the low-risk group.

Previous studies have confirmed TMB as a novel biomarker that can predict response to tumor immunotherapy^77^. Since overall neoantigen burden is difficult to measure and TMB is easily detected and used to assess neoantigen burden, has been shown to be an indicator of clinical benefit or prognostic factor with the potential to predict ICI response^78^. We found a positive relevance between TMB and riskscore. The tumor suppressor gene CDH1 had a higher mutation level in low-risk group, while the oncogene TP53 had a higher mutation frequency in high-risk group. All of these findings emphasize the relationship between TMB and TME, and suggest that the TME-related prognostic signature can more effectively predict prognosis and immunotherapy response.

Our study demonstrates a TME-related prognostic signature in the transcriptome through existing common tumor databases. This novel TME-related prognostic signature may facilitate more personalized prognostic prediction in BRCA patients and serves as a potential biomarker and therapeutic target. Inevitably, our study has some notable limitations. First, all the data we used were retrospective, and the efficacy of TME-related prognosis signature needs to be further verified in prospective studies. Then, we have not conducted any experimental studies on each gene to learn more about it and the underlying mechanism. Finally, we should include more clinical parameters into the TME-related prognosis signature scoring system, so as to improve the accuracy of prediction and provide higher reference value for clinical treatment.

## Methods

### Data collection

The RNA-sequencing datasets of 113 normal samples and 1109 BRCA samples were obtained from TCGA (https://portal.gdc.cancer.gov). Clinical data (including 1091 samples), DNA methylation data (including 890 samples) and subtype information (including 187 Basal subtypes, 77 Her2 subtypes, 552 LumA subtypes and 202 LumB subtypes) for BRCA samples were collected from the TCGA portal (Supplementary Table 1). The cbioportal for Cancer Genomics was where we downloaded METABRIC RNA-sequencing data of 1904 samples, mutation data and related clinical data (https://www.cbioportal.org/datasets). Microarray data and clinical information for all validation datasets were downloaded from the GEO database (https://www.ncbi.nlm.nih.gov/geo/). Among them, GSE21653 contains 252 samples, and GSE58812 has 107 samples^79,80^. The TCGA-BRCA somatic mutation data was also obtained from TCGA. We obtained a transcriptome dataset of breast cancer from the ICGC database, including 99 breast cancer samples (https://dcc.icgc.org/). And we obtained a transcriptome dataset of breast cancer from Krug, Karsten *et al*., which included 122 breast cancer samples^81^. We obtained the breast cancer protein dataset PDC000173 from Proteomic Data Commons (PDC, https://pdc.cancer.gov/pdc/), including 105 BRCA samples^28^.

### TME Construction

Based on RNA-seq data from the TCGA-BRCA cohort, we used the ESTIMATE algorithm to calculate the estimate score, stromal score, and immune score to characterize the TME^82^. The stromal score and immune score represent the infiltration levels of stromal cells and immune cells in tumor tissue, respectively. The estimate score was the combination of stromal score and immune score to represent the measurement of tumor purity^83^. A lower estimate score, stromal score and immune score represent higher tumor purity and lower degree of infiltration of stromal cells and immune cells in tumor tissue, respectively.

### Generation of DEGs

We classified 1109 tumor samples into high and low groups based on the median of immune score and stromal score, respectively. DEGs were generated by comparing high-group samples with low-group samples using the DEseq2 package. Adj. p < 0.05 and |Log2FC| > 1 were used as the threshold for screening DEGs.

### Establishment and validation of the TME-related prognosis signature

Univariate Cox regression analysis and multivariate Cox regression analysis were used to screen the candidate genes related to prognosis in TME DEGs. The LASSO Cox regression model reduces redundant genes by reducing the dimensionality of the data. Then, the candidate genes were then subjected to LASSO regression analysis by the R package “glmnet” and 10-fold cross validation. Risk score of each BRCA patient was calculated by using key TME-related genes corresponding regression coefficients and expression levels. The formula is ${\rm{Riskscore}}={\sum }_{{\rm{i}}=1}^{{\rm{n}}}({{\rm{coef}}}_{{\rm{i}}}\times {\rm{\exp }}{{\rm{r}}}_{{\rm{i}}})$. Here, *coef*_*i*_ is the Cox coefficient of *gene*_*i*_ and *expr*_*i*_ is the expression of the *gene*_*i*_. Patients were divided into high-risk and low-risk groups on the basis of the median risk score. Kaplan-Meier analysis was performed using the R package “survival” to assess the overall survival of patients in different groups.

### Gene set variation analysis (GSVA)

GSVA is a method to estimate the enrichment level of a gene set in each sample through the expression data set of the sample. The gene set files of “h all.v7.4.symbols” including 50 key gene sets were obtained from the MSigDB of Broad Institute (http://www.gsea-msigdb.org/). We used the package “limma” and “GSVA” in R to recognized the biological processes of co-activation or inhibition in different groups, with p < 0.05 and |logFC| > 0.1 is the threshold value. We conduct the gene enrichment analysis in terms of gene ontology (GO) and KEGG using the DEGs grouped based on different scores through the package “clusterProfiler” and “enrichplot”.

### Construction and validation of a predictive nomogram

Univariate and multivariate Cox regression analyses were performed on the risk score and other clinicopathological characteristics to identify the independent factors affecting the prognosis, which were visualized by the software package “forestplot” in R. Then, the confirmed prognostic factors and the software packages “RMS” and “regplot” in R were then used to develop a nomogram prediction model for predicting 10-to 20-year mortality in BRCA patients. Next, calibration Curves and decision Curve Analysis (DCA) were employed to estimate the predictive effect of Nomogram and its clinical potential.

### Mutation enrichment analysis in the high-risk group and low-risk group

The permutation test was employed to assess whether the mutant gene was enriched in the high-risk group and low-risk group. Specifically, patients with BRCA were divided into high-risk group and low-risk group. The mutation frequency of the mutant gene G was H in the high-risk group, and was L in the low-risk group. The observed enrichment ratio of the mutant gene G for the high-risk group and the low-risk group could be denoted as high-risk enrichment ratio = H/L and low-risk enrichment ratio = L/H, respectively. We randomly selected high-risk group samples and low-risk group samples from the background mutation set of BRCA samples and calculated the mutation frequency of mutant gene G, and then calculated the random high-risk enrichment ratio and low-risk enrichment ratio of gene G. After repeating the procedure 1000 times, we would obtain a P-value of mutant gene enrichment in the high-risk group by dividing the times when the random high-risk enrichment ratio was greater than the observed high-risk enrichment ratio (H/L) by 1,000. The P-values of mutant gene enrichment in the low-risk group were estimated using the similar methods^84,85^.

### Expression abundance of immune cell in TME

XCell uses curve fitting to make linear comparisons of cell types, from which the types of immune cells and stromal cells are inferred^86^. The xCell R software package was used to evaluate the expression abundance of 22 immune cells in all samples. CIBERSORT is an analytical method that uses gene expression data to estimate the abundance of immune cell types in tumor samples^87^. The TIMER algorithm was used to estimate the abundance of 6 kinds of immunoinfiltrating cells in breast cancer samples^88^.

### Evaluation of the immunological therapy response

The predictive efficiency of prognostic features for response to BRCA ICIs was evaluated using the Tumor Immune Dysfunction and Exclusion algorithm (TIDE). A high TIDE score was associated with a greater likelihood of immunotherapy non-responder, while a low TIDE score was the opposite.

### Statistical analyses

The statistical significance for the two sets of variables that fit a normal distribution was estimated by unpaired Student’s t tests. Moreover, the Kruskal-Wallis test was used for variables with more than two groups. The Kruskal-Wallis test and Wilcoxon rank sum test were applied to analyze correlations between the risk score and clinic-pathological parameters. Correlation analysis between two groups of variables was used spearman correlation coefficient.

## Supplementary information

Supplementary information

## Data Availability

The data that support the findings of this study are available from the TCGA-BRCA (https://portal.gdc.cancer.gov/projects/TCGA-BRCA). The additional validation data in this article were obtained from METABRIC (Breast Cancer, Nature 2012 & Nat Commun 2016) (http://www.cbioportal.org/datasets), ICGC (https://dcc.icgc.org/), Krug, Karsten *et al*.^81^, Proteomic Data Commons (https://pdc.cancer.gov/pdc/) and Gene Expression Omnibus (GEO) with the accession number GSE21653^79,89^ and GSE58812^80,90^. The analysis results associated with this paper is available on Github (https://github.com/wangliTeam/data-and-code).

## Code availability

The R code used in the analysis of the data is available on GitHub (https://github.com/wangliTeam/data-and-code).
