# Supplementary material for: Identifying tumour microenvironment-related signature that correlates with prognosis and immunotherapy response in breast cancer
Source: Sci Data. 2023 Mar 3;10:119. doi: 10.1038/s41597-023-02032-2 (PMC9984471; doi:10.1038/s41597-023-02032-2)
Supplement: Supplementary file 1 — Supplementary information [file 41597_2023_2032_MOESM1_ESM.pdf]

SUPPLEMENTARY INFORMATION

SUPPLEMENTARY INFORMATION 1

SUPPLEMENTARY FIGURES 2

Supplementary Figure 1 2

Supplementary Figure 2 3

Supplementary Figure 3 4

Supplementary Figure 4 5

Supplementary Figure 5 6

Supplementary Figure 6 7

Supplementary Figure 7 8

Supplementary Figure 8 9

Supplementary Figure 9 10

Supplementary Table 1 11

Supplementary Table 2 11

Supplementary Table 3 11

SUPPLEMENTARY FIGURES

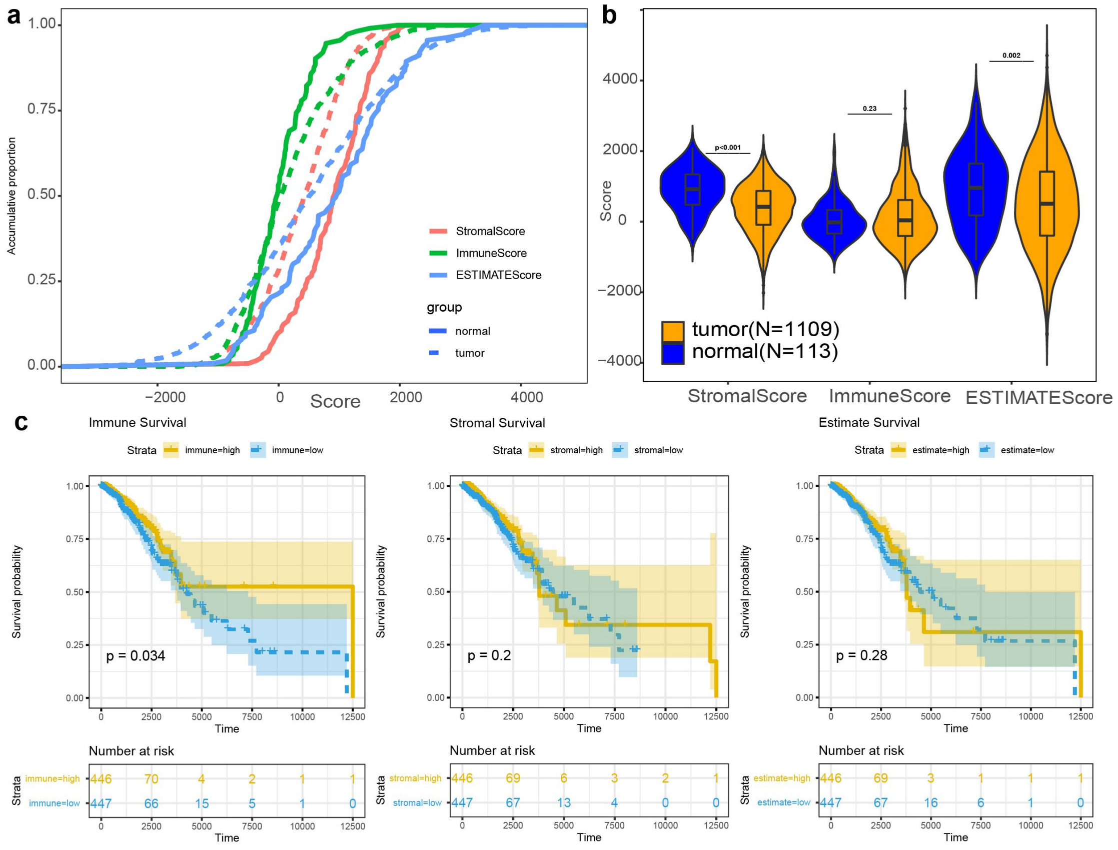

Figure S1. The tumor microenvironment (TME) in TCGA-BRCA cohort. (a, b) Comparison of the distributions of estimate scores, immune scores, and stromal scores between tumor and normal cohorts. (c) Kaplan-Meier curves show the independent relevance between overall survival time and immune scores, stromal scores and estimate scores.

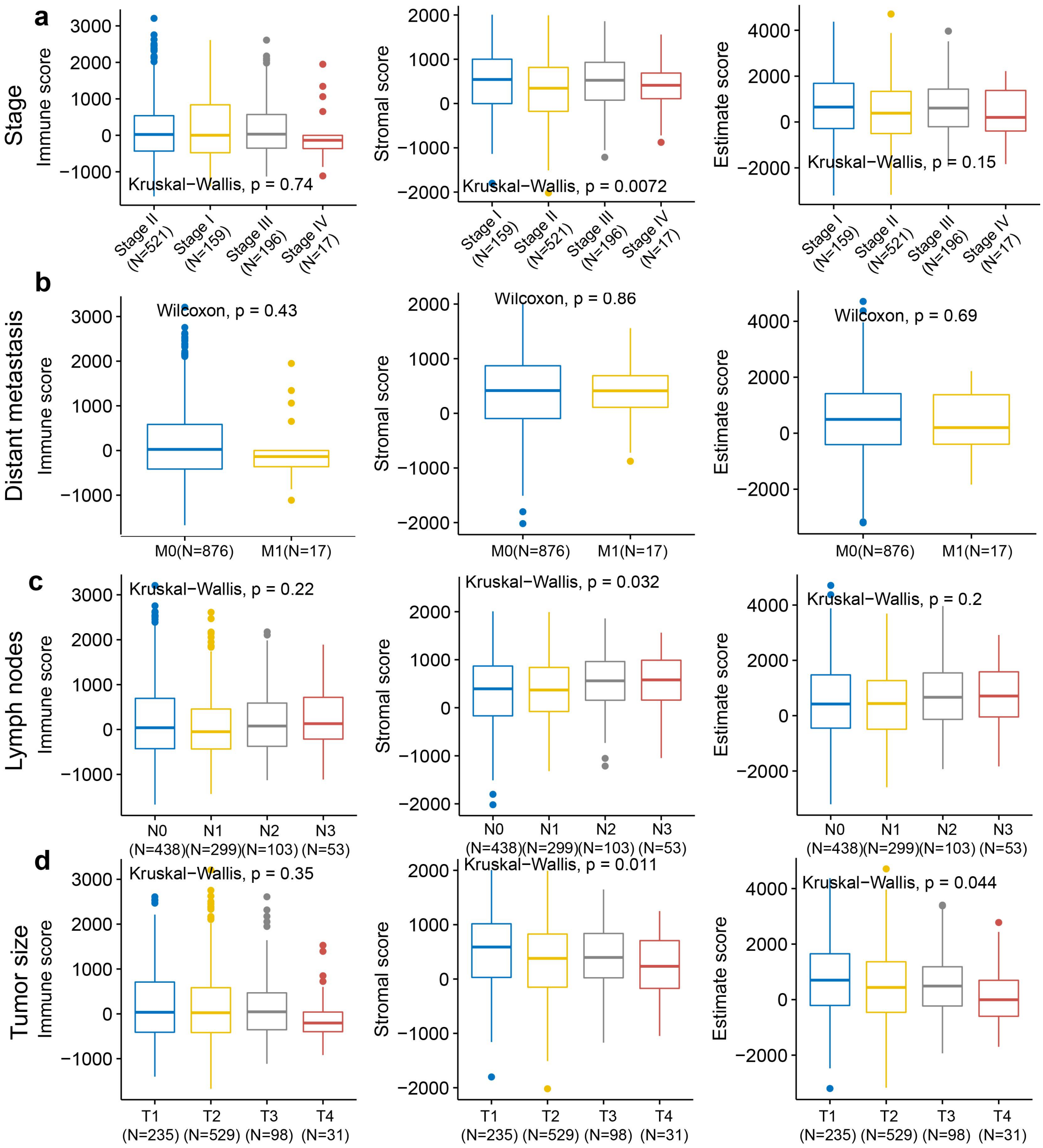

Figure S2. Distribution of ImmuneScore, StromalScore, and ESTIMATEScore in stage (a), M classification (b), N classification (c) and T classification (d).

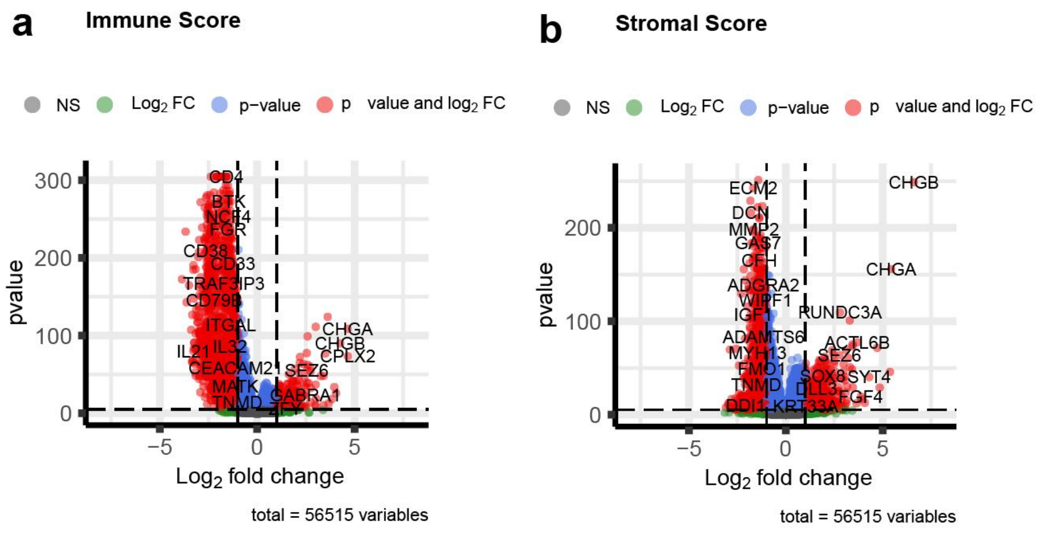

Figure S3. Differential gene expression profiles in breast cancer. volcano plot of differential genes in the high vs low stromal score groups (a) and in the high vs low immune score groups (b).

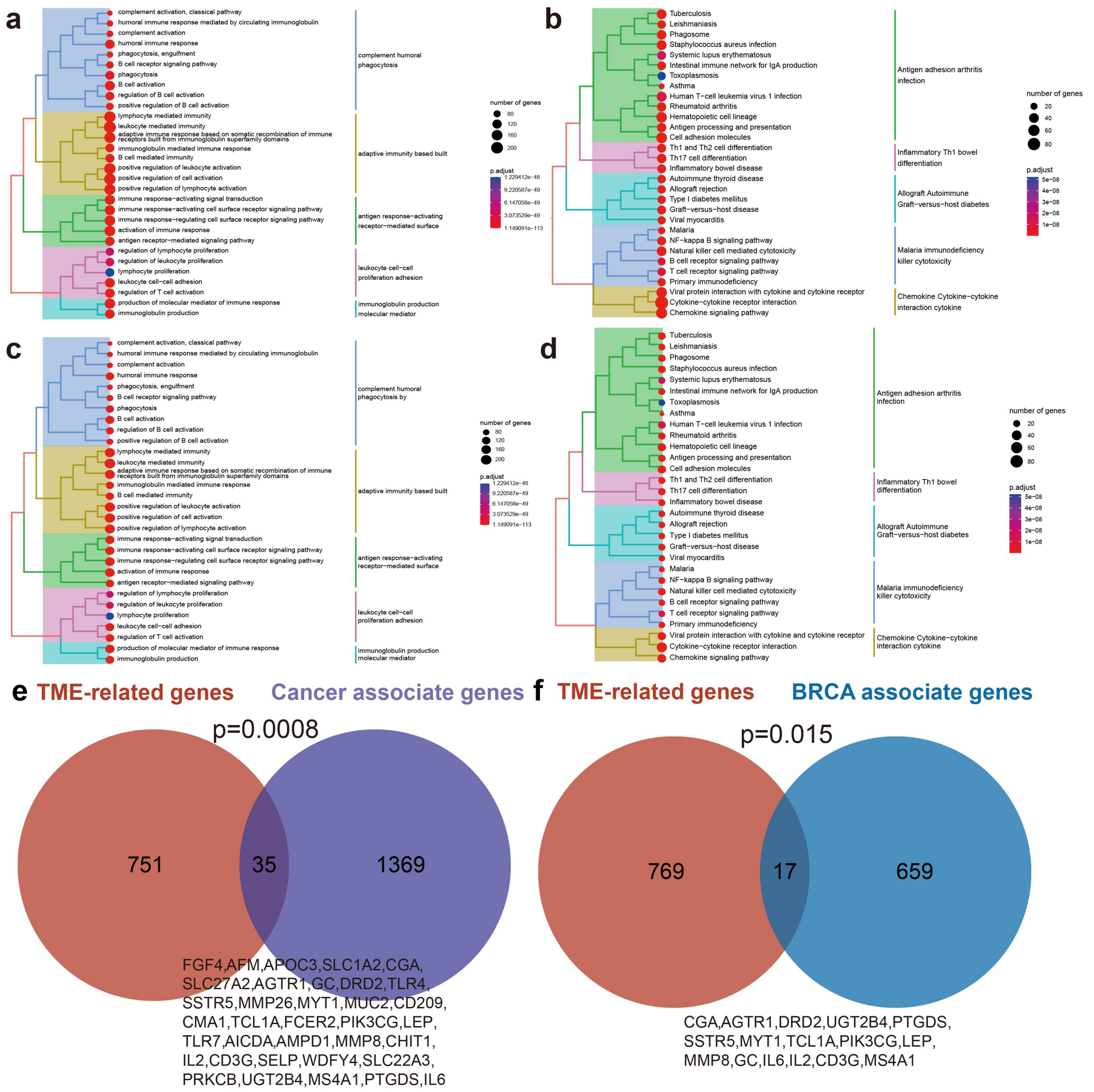

Figure S4. Gene enrichment analysis based on different scores. (a) GO enrichment analysis for the immune score difference gene. (b) KEGG enrichment analysis for the immune score difference gene. (c) GO enrichment analysis for the stromal score difference gene. (d) KEGG enrichment analysis for the stromal score difference gene. The size of the dots indicates the number of clustered genes and the color of the dots signifies the adjusted p-value of enrichment. (e) Venn diagram shows the common genes of TME-related genes and cancer-associated genes. (f) Venn diagram shows the common genes of TME-related genes and BRCA-associated genes.

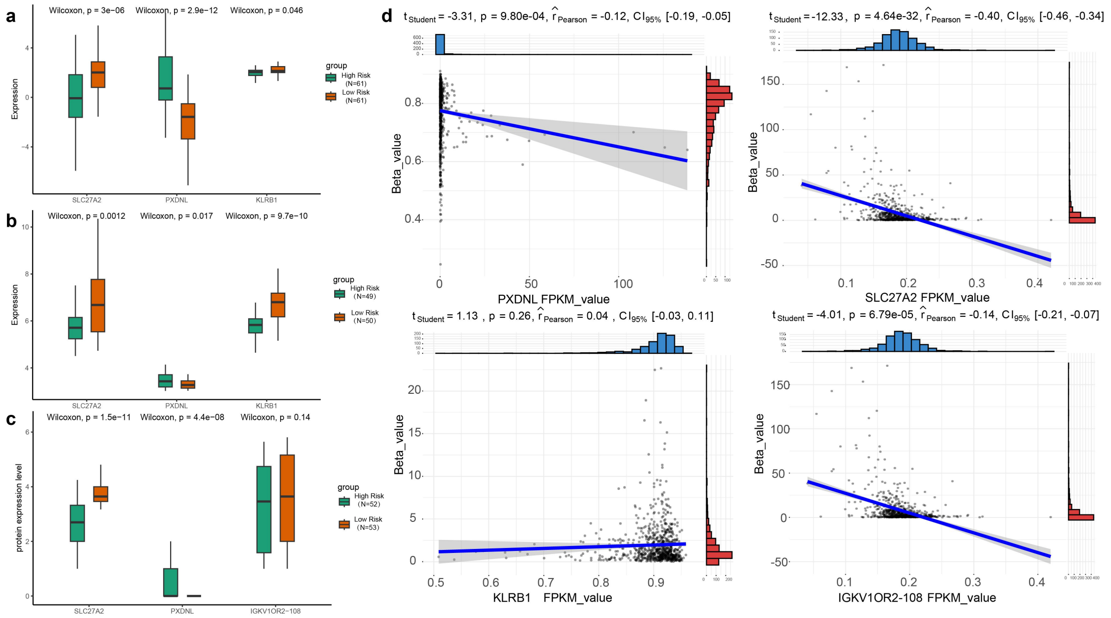

Figure S5: Key TME-related genes in relation to risk score and DNA methylation. (a, b) Differential expression of key TME-related genes between high- and low-risk score groups. (c) Differences in protein expression levels of key TME-related genes between high- and low-risk score groups. (d) Key TME-related genes correlation with DNA methylation.

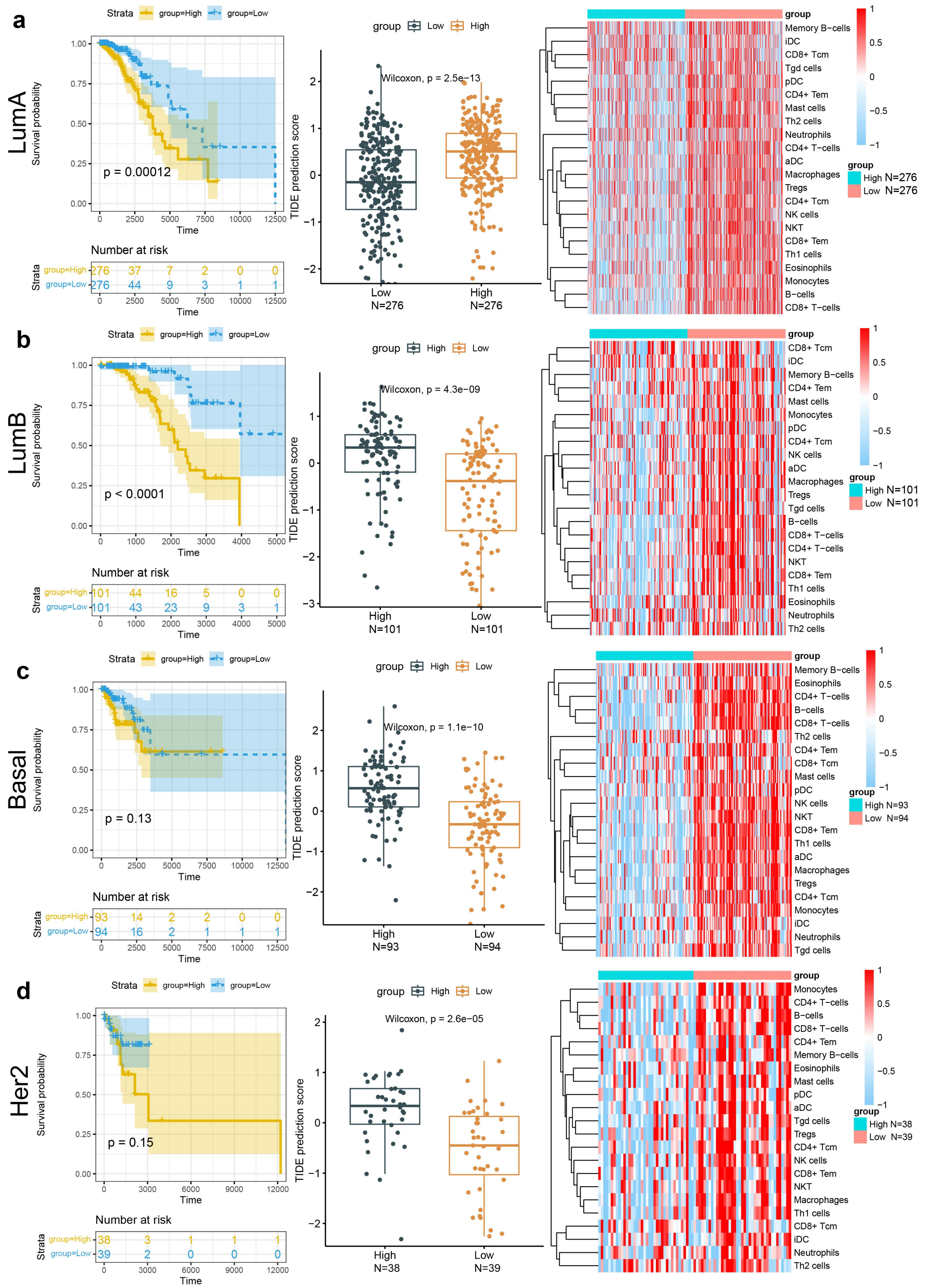

Figure S6: Prognostic signature of different subtypes of breast cancer. (a) Changes in prognostic signature, TIDE score, and degree of immune cell infiltration in the LumA subtype based on the risk score. (b) Changes in prognostic signature, TIDE score, and degree of immune cell infiltration in the LumB subtype based on the

risk score. (c) Changes in prognostic signature, TIDE score, and degree of immune cell infiltration in the Basal subtype based on the risk score. (d) Changes in prognostic signature, TIDE score, and degree of immune cell infiltration in the Her2 subtype based on the risk score.

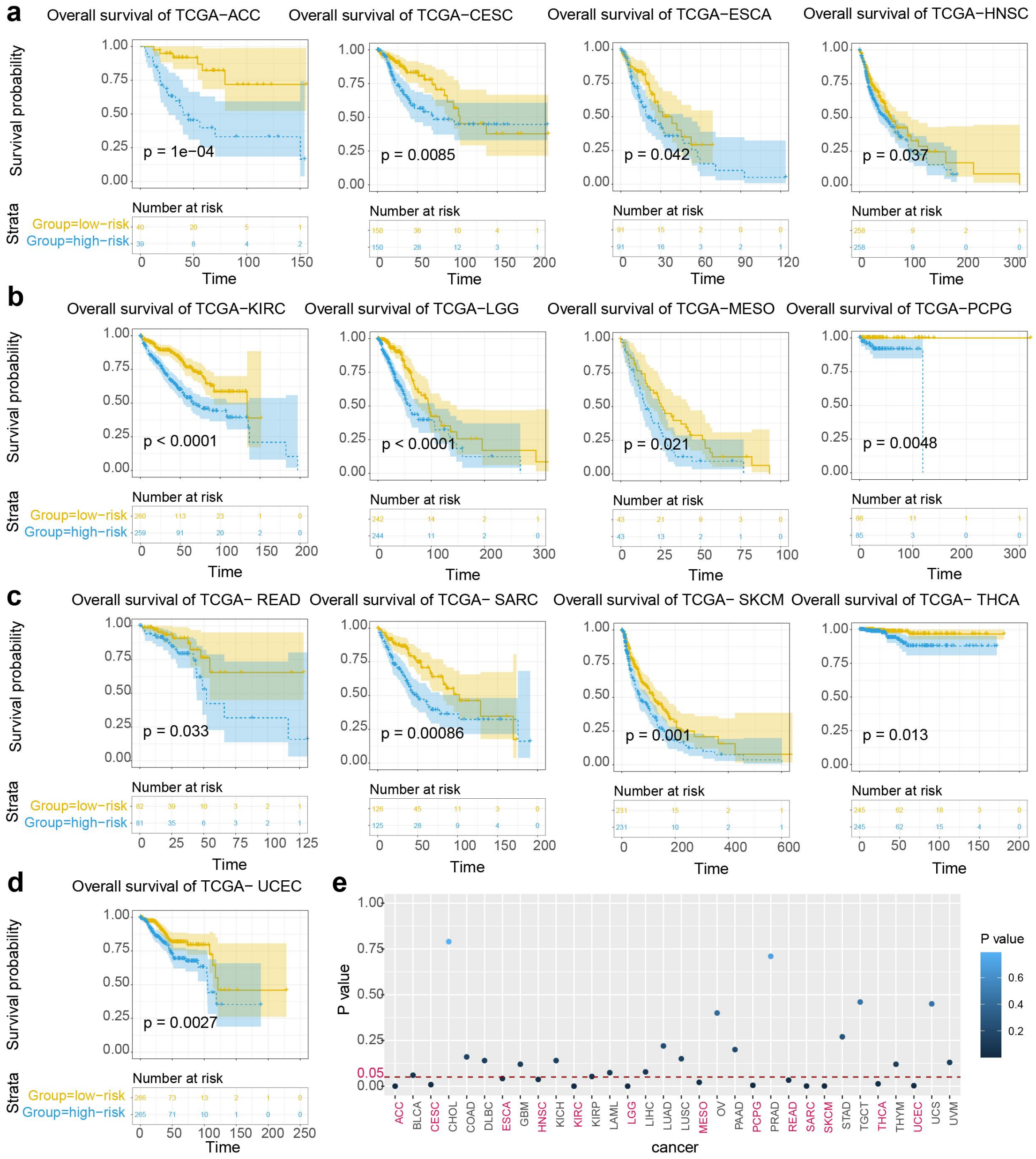

Figure S7: Survival analysis of six TME-related genes in pan-cancer. (a-d) Kaplan - Meier plot curves and risk score of overall survival based on risk scores of the six TME-related genes in 13 cancer types including ACC, CESC, ESCA, HNSC (a), KIRC, LGG, MESO, PCPG (b), READ, SARC, SKCM, THCA (c) and UCEC (d)

from TCGA. The P-values was calculated using the log - rank test. (e) A total of 32 types of cancer were analyzed. The red dotted line represents a p value equal to 0.05. Filled colours from dark blue to red represent P-values.

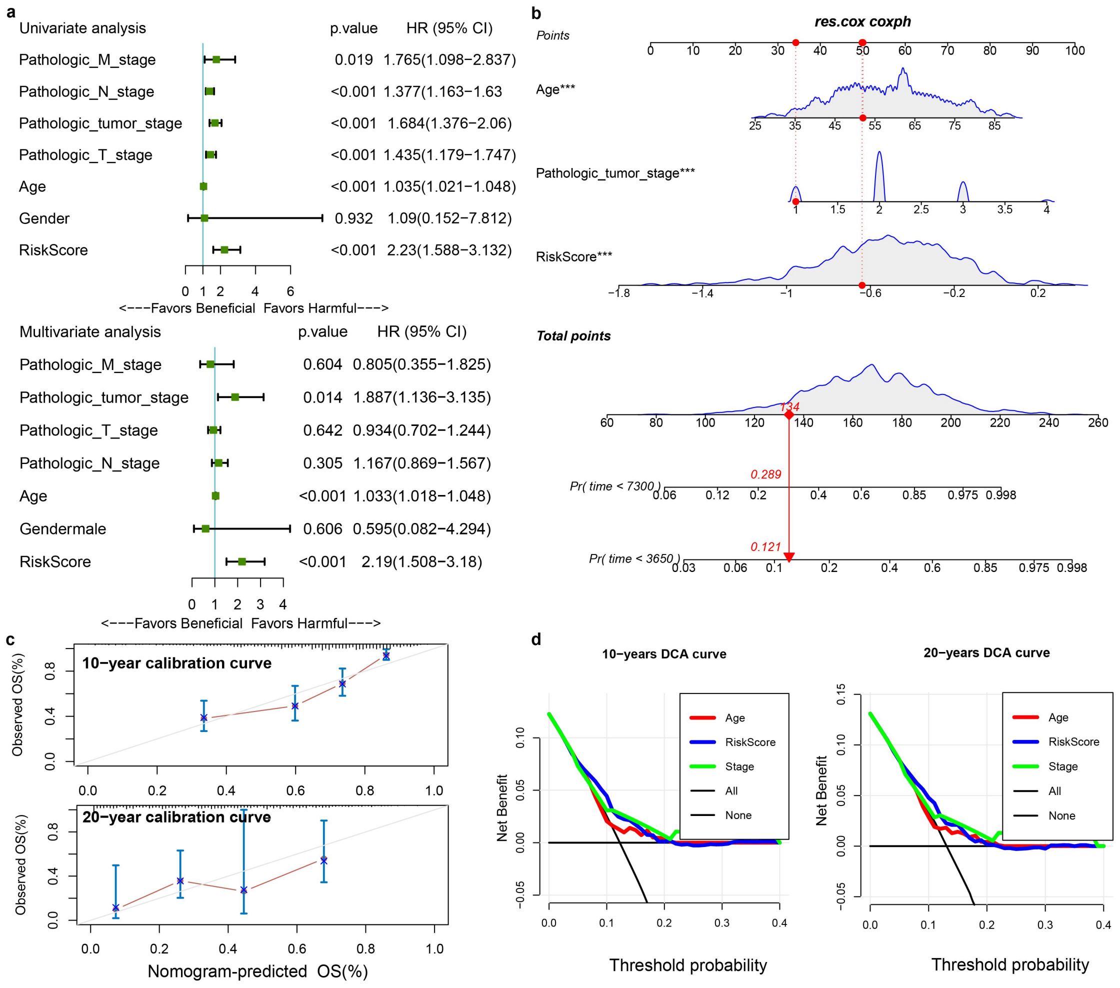

Figure S8. The prognostic signature acting as an independent prognostic factor in BRCA. (a) Univariate and multivariate COX analyses of the prognostic signature and clinic-pathological features were recorded in the forest plot. The green squares on the transverse lines represent the HR, and the black transverse lines rep resent the 95% CI. The P value and 95% CI for each clinical feature are displayed in detail. (b) Nomograms for predicting the probability of patient mortality at 10- or 20-year OS based on risk scores of the prognostic signature, age and tumor stage. (c) Calibration curves of the nomogram for predicting the probability of OS at 10-, and 20-years. (d) Decision curve analyses (DCA) curve of the nomogram based on TMERS for

10-years and 20-years OS prediction.

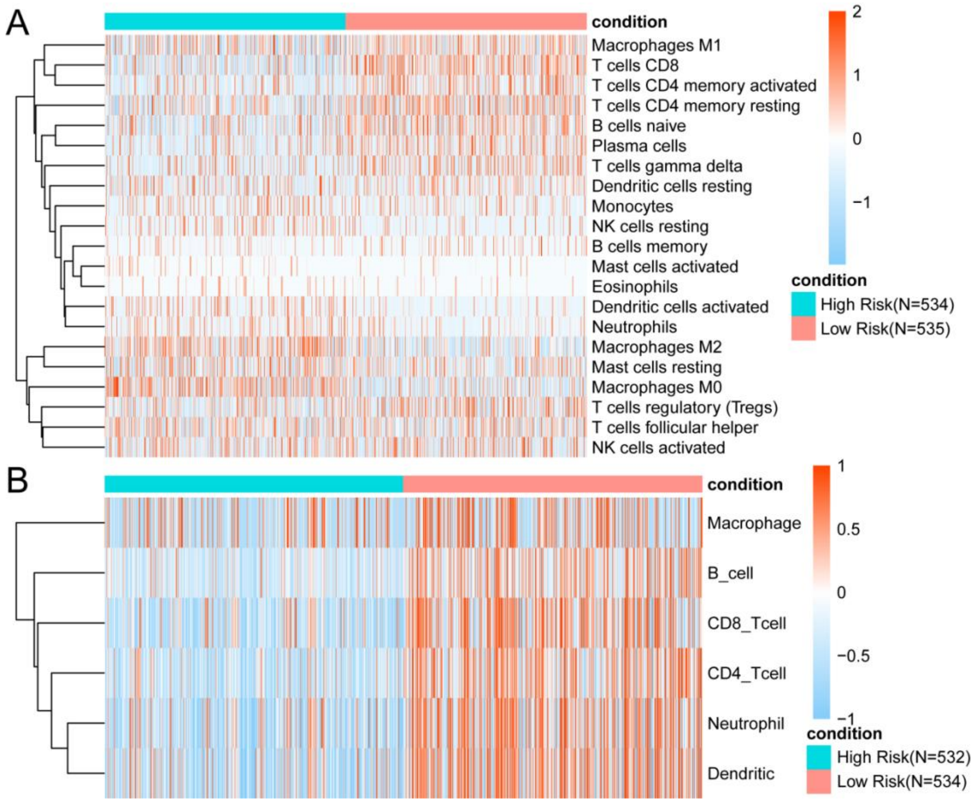

Figure S9: The prognostic signature correlated with immune cell infiltration in BRCA. (a) Estimating the abundance of 21 types of immune cells in the high- and low-risk score groups in TCGA-BRCA cohort using CIBERSORT. (b) Estimating the abundance of 6 types of immune cells in the high- and low-risk score groups in TCGA-BRCA cohort using TIMER.

Supplementary Table 1: Data Sources and Data Usage

Data From	Sample	Data Type	Data Use
TCGA	113 samples	Normal RNA-seq data	Control samples
TCGA	1109 samples	BRCA RNA-seq data	TME construction、Difference analysis
TCGA	1091 samples	BRCA Clinical data	Survival analysis
TCGA	985 samples	BRCA mutation data	Mutation analysis
TCGA	890 samples	DNA methylation data	Methylation analysis
METABRIC	1904 samples	RNA-seq data+clinical data	independent validation cohorts
METABRIC	2355 samples	mutation data	independent validation cohorts
GEO(GSE21653)	252 samples	Microarray data+clinical data	independent validation cohorts
GEO(GSE58812)	107 samples	Microarray data+clinical data	independent validation cohorts
ICGC	99 samples	Microarray data	independent validation cohorts
PDC	105 samples	Proteome data	independent validation cohorts
Krug, Karsten et al.	122 samples	RNA-seq data	independent validation cohorts

Supplement Table 2: Genes screened by different machine learning algorithms and their concordance rate

	SLC27A2	PXDNL	LINC02038	KLRB1	IGKV1OR2-108	IGHV1-12	total
Lasso	√	√	√	√	√	√	6
RSF	√	√	√	√	√	√	18
Ridge	√	√	√	√	√	√	33
Enet	√	√	√	√			13
stepwiseCox	√	√		√			8
plsRcox	√			√			11
SuperPC		√					1

Supplement Table 3: Potential prognostic molecules identified by multiple machine learning algorithms

Lasso	SLC27A2, PXDNL, LINC02038, KLRB1, IGKV1OR2-108, IGHV1-12
RSF	SLC27A2, PXDNL, LINC02038, FP671120.7, KLRB1, TESPA1, CD1E, IGHV1OR15-9, DTHD1, SPN, IGHA1, IGKV1OR2-108, IGKV1-6, IGHV3-60, IGHV1-12, IGHV3-42, IGHV3OR16-13, AC134879.2
Ridge	SLC27A2, PXDNL, ELAVL3, LINC02038, FP671120.7, CD40LG, KLRB1, JCHAIN, TESPA1, CD1E, CCL19, IGHV1OR15-9, DTHD1, SPN, IGKV1OR2-3, TRAV12-3, TRAV27, IGHA1, IGHV3-15, IGHV3-23, IGHV3-53, IGKV1OR2-108, TRBV29-1, IGKV1-6, IGKV2D-40, IGHV1-14, IGHV3-60, IGHV3-19, IGHV1-12, IGHV3-42, IGHV3OR16-13, AC134879.2, TRBV7-9
Enet	SLC27A2, PXDNL, ELAVL3, LINC02038, FP671120.7, KLRB1, CD1E, CCL19, TRAV12-3, TRAV27, IGHV3-15, TRBV29-1, IGKV1-6
stepwiseCox	SLC27A2, PXDNL, ELAVL3, KLRB1, SPN, TRAV27, IGHA1, IGHV1-14
plsRcox	PXDNL, ELAVL3, CD40LG, KLRB1, TESPA1, CCL19, SPN, TRAV12.3, TRAV27, TRBV29.1, TRBV7.9
SuperPC	PXDNL
